# Comparison of Lipid Measurements by Clinical Chemistry and NMR Spectroscopy

**DOI:** 10.3390/diagnostics16010028

**Published:** 2025-12-22

**Authors:** Nazlıhan Tekin, Neslihan Yıldırım Saral, Aysun Toker, Furkan Şahin, Ahmet Tarık Baykal, Mustafa Serteser

**Affiliations:** 1 Acibadem Labmed Clinical Laboratories, Istanbul 34752, Turkey; nazlihanyildirim@hotmail.com (N.T.); neslihan.saral@acibademlabmed.com.tr (N.Y.S.); aysun.toker@acibademlabmed.com.tr (A.T.); furkan.sahin1@live.acibadem.edu.tr (F.Ş.); 2 Department of Biochemistry and Molecular Biology, Graduate School of Health Sciences, Acibadem Mehmet Ali Aydinlar University, Istanbul 34752, Turkey; 3 Department of Medical Biochemistry, School of Medicine, Acibadem Mehmet Ali Aydinlar University, Istanbul 34752, Turkey

**Keywords:** NMR, lipids, lipoproteins, clinical chemistry, freezing-thawing

## Abstract

**Background/Objectives**: Triglyceride (TG), Total Cholesterol (TC), HDL cholesterol (HDL-C), and LDL cholesterol (LDL-C) levels are commonly tested routine lipid profiles (RLPs) for assessing cardiovascular disease (CVD) risk. While lipid levels are typically measured by using standard clinical chemistry tests in routine practice, Nuclear Magnetic Resonance (NMR) spectroscopy has recently been explored for its ability to determine lipid levels under clinical settings. This study aims to compare RLP and NMR analysis using 17,337 fresh serum samples. Additionally, it investigates the impacts of freezing–thawing on these parameters in 9559 frozen samples. **Methods**: RLP was performed by employing the Siemens Dimension clinical chemistry system. Furthermore, the lipid contents of the fresh and frozen serum samples were evaluated across different concentration ranges. **Results**: Lipid parameters of fresh samples ascertained with RLP and NMR were strongly correlated (r ≥ 0.93). Analysis with frozen samples revealed that the correlation between lipid measurements decreased below r ≤ 0.86, except for TG (r = 0.97). Additionally, at different concentration ranges, the lower-level ranges for all lipid parameters in both fresh and frozen samples exhibited weaker correlations. **Conclusions**: This study demonstrates that NMR spectroscopy is a reliable, rapid, chemical-free method for lipid analysis in fresh samples. However, in frozen samples, relying on NMR to support RLP offers a less reliable approach for lipid measurement.

## 1. Introduction

Lipids are essential for many cellular processes; it is involved in the formation of a membrane bilayer, provides a hydrophobic environment for membrane proteins and facilitates their interactions, serves as a secondary energy source, and participates in cell division, growth, and death [1]. Abnormalities or defects in lipid metabolism have been linked to numerous disorders, ranging from metabolic diseases like diabetes mellitus and obesity to autoimmune diseases such as multiple sclerosis (MS) and cardiovascular disease (CVD), and to more complex conditions, including Alzheimer’s disease and cancer [2]. It is crucial to investigate lipid levels, especially in clinical samples, to better understand their effects on disease progression and the mechanisms that regulate the responses of patients to various treatments.

Since they are insoluble in water, lipids such as cholesterol and triglyceride (TG) are carried through blood circulation conjugated with proteins called lipoproteins [3]. There are five main types of lipoproteins: chylomicrons, very-low-density lipoproteins (VLDLs), low-density lipoproteins (LDLs), intermediate-density lipoproteins (IDLs), and high-density lipoproteins (HDLs) [4]. These lipoproteins are essential for the absorption of dietary fats by the small intestines and for the movement of fats from the intestines to the liver and peripheral tissues and back to the intestines [5]. It is known that LDL and HDL are associated with CVD [6].

A routine lipid profile (RLP) includes the measurement of TG, Total Cholesterol (TC), high-density lipoprotein cholesterol (HDL-C), and low-density lipoprotein cholesterol (LDL-C), which contain TG and cholesterol. The laboratory testing of these parameters is common, since clinical practice guidelines recommend their routine use for assessing CVD risk [7]. RLP is typically performed in clinical laboratories using automated enzymatic or colorimetric assays. Although these methods are quick and sensitive, they require several chemicals. Moreover, they are vulnerable to endogenous interferences within the sample, such as lipemia, hemolysis, and icterus, since they employ spectrophotometric detection [7]. In contrast, Nuclear Magnetic Resonance (NMR) spectroscopy offers an alternative high-throughput approach to determine the plasma lipid profile. Its benefits include less susceptibility to analytical interferences and no need for specific reagents. Additionally, with this technique, the concentration or average size of each lipoprotein class—VLDL, LDL, and HDL—can be ascertained simultaneously. Conversely, standard lipid tests only measure the cholesterol or TG content within lipoproteins. However, the cholesterol content of lipoproteins varies with the metabolic state of the individual; for example, discrepancies can occur between the numbers of LDL-C and low-density lipoprotein particle number (LDL-P). The literature shows that LDL-P is more strongly associated with CVD than LDL-C [8]. Therefore, NMR technology has a notable advantage over traditional lipid tests. Thus, this study comparatively analyzed the lipid values obtained via NMR and standard clinical chemistry methods.

Serum or plasma lipid analysis is routine for the clinical diagnosis and treatment of various diseases and is also used in medical research to identify disease mechanisms and biomarker discovery, termed as lipidomics [2]. To perform clinical research, biobanks collect and store plasma and serum samples. These samples in biobanks are usually stored in liquid nitrogen tanks (−196 °C) or in −80 °C freezers prior to analysis [9]. However, due to a large sample size and limited storage space in biobanks, the samples are subjected to repeated freeze–thaw cycles for routine clinical analysis and research studies [10]. The NMR method is used to reduce the number of freezing–thawing cycles in biobanks for lipidomics or routine clinical chemistry. Several studies have demonstrated that the serum lipoprotein and metabolite levels change with the number of freeze–thaw cycles [11,12,13]. However, these studies did not report a comparison of clinical chemistry tests and the NMR method.

In light of the above observations, the current study aims to compare the RLP and NMR-derived values of TC, TGs, HDL-C, and LDL-C in fresh serum samples. Thus, we suggest an effective method using NMR to measure lipid molecules as an alternative to RLP. Furthermore, this study provides data showing whether NMR can be a suitable alternative method in freeze–thaw procedures in RLP.

## 2. Materials and Methods

### 2.1. Collection of Samples

In total, 17,337 (from 9588 males and 7749 females in the range of 18–75 old) and 9559 (from 5246 males and 4313 females) blood samples were collected in tubes with non-additives for comparing RLP- and NMR-based measurements, as well as the effects of freeze–thaw cycles, respectively. The tubes were centrifuged at 1800× *g* for 10 min, and the sera obtained were aliquoted.

The aliquots for comparison between RLP and NMR in the fresh serum samples were stored at +4 °C until analysis (intra-day). To determine the effect of freeze–thaw cycles, the aliquots for RLP were stored at +4 °C until analysis (intra-day), while the aliquots for NMR analysis were frozen in −80 °C until analysis (in two weeks). Before analysis, the samples were thawed at +4 °C.

The study protocol received approval from the Institutional Ethical Review Board of Acibadem Mehmet Ali Aydınlar University, İstanbul, Turkiye (2024-13/538), per the World Medical Association Declaration of Helsinki.

### 2.2. Sample Preparation and Analysis

Clinical chemistry analysis for standard lipid tests was conducted on Siemens Dimension^®^ EXL™ using the Siemens Dimension clinical chemistry analyzer (Siemens Healthineers, Erlangen, Germany).

For NMR analysis of fresh serum samples, 400 µL of serum was mixed with 400 µL of plasma buffer containing 20% deuterium oxide, 0.075 M sodium monophosphate, 4.6 mM sodium 3-(trimethylsilyl)-2,2,3,3-tetradeuteropropionate (TSP), and 0.04% NaN_3_ (Bruker BioSpin GmbH, Ettlingen, Germany). The mixture was transferred into 5 mm SampleJet NMR tubes (Bruker BioSpin, Ettlingen, Germany) [11]. Lipid profiles were automatically analyzed using the commercial Bruker IVDr Lipoprotein Subclass Analysis (B.I.LISA) method (Bruker BioSpin) [13].

For the NMR analysis of the frozen serum samples, aliquots were thawed at +4 °C and rotated for 4 min. Then, the samples were prepared by employing the steps mentioned above.

#### NMRData Acquisition

NMR data acquisition was performed using a Bruker Avance Neo 600 MHz IVDr NMR spectrometer (Bruker BioSpin, Ettlingen, Germany) equipped with a 5 mm BBI Probe, the Bruker SampleJet robotic system (Bruker), and Topspin software 4.3.0 (Bruker). Nuclear Overhauser Enhancement Spectroscopy (NOESY) with a “noesygppr1d” pulse sequence was utilized to acquire the 1H NMR spectra, recorded at 310 K. In addition, the main parameters were set as follows: total scans, 32; spectral width, 30 ppm; dummy scans, 4; relaxation delay, 4 s; and total acquisition time, 4 min 4 s [13].

### 2.3. Statistical Analysis

The clinical chemistry and NMR results were statistically evaluated using the R software program version 4.5.0. The results were compared using Spearman correlation analysis. In this test, Spearman coefficients (r) ≥ 0.75 and (r) ≥ 0.2 were identified as strong/moderate and weak correlations, respectively [14]. The normality distribution of the lipid results was confirmed by performing the Shapiro–Wilk test with <5000 samples as this test yields better results with this number [15]. A *t*-test was applied to examine the statistical significance of the differences between clinical chemistry and NMR values at *p* < 0.05.

## 3. Results

### 3.1. Comparison Between RLP and NMR Analysis for TG, TC, HDL-C, and LDL-C in Fresh Serum Samples

In this study, TG, TC, HDL-C, and LDL-C measurements in fresh serum samples were performed using a standard RLP and the NMR method. Furthermore, all lipid parameters in fresh serum samples were evaluated using different level ranges, and the sample sizes for these ranges are demonstrated in Table 1. Reference range values were determined according to routine lipid panel ranges of the Acibadem Health Group for adults. The applied statistical analysis results, such as correlations and *t*-tests, are summarized in Table 2.

**Figure 1:**
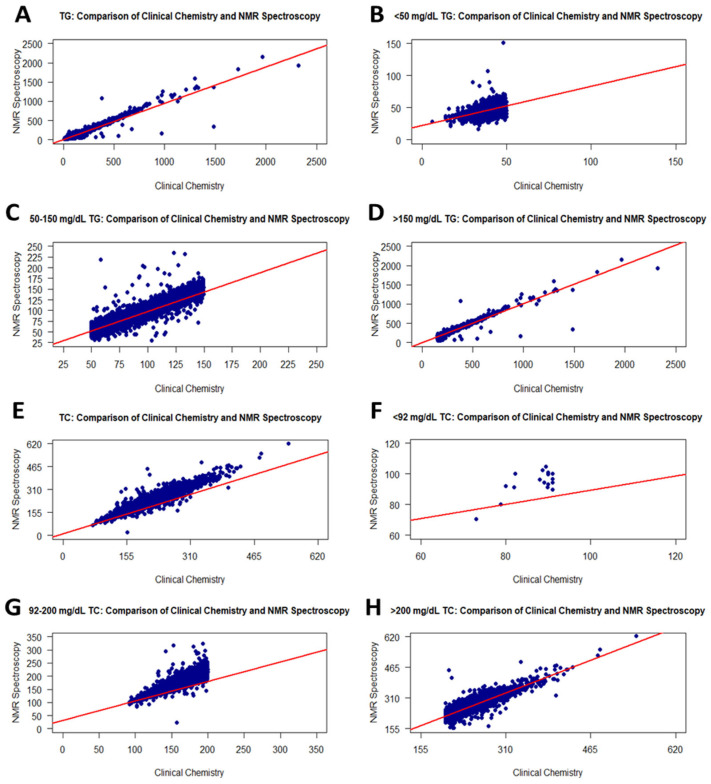
Graphs showing the correlation between RLP and the NMR method for TG and TC values. Scatter plots of correlations for all TG and TC values (**A**,**E**): <50 mg/dL TG (**B**), 50–150 mg/dL TG (**C**), >150 mg/dL TG (**D**), <92 mg/dL TC (**F**), 92–200 mg/dL TC (**G**), and >200 mg/dL TC (**H**).

TG measurements were conducted on a total of 17,334 fresh serum samples using both standard RLP and the NMR method. The results from the NMR and RLP groups did not follow a normal distribution according to the Shapiro–Wilk test, with a *p*-value of <0.001. Additionally, a *t*-test analysis revealed statistically significant differences between the NMR and RLP-derived values, with a *p*-value of <0.0001 (Table 2). Regarding correlation analysis, TG values obtained employing the RLP and NMR methods were robustly associated with each other, with r and *p*-values of 0.98 and <0.0001, respectively (Figure 1A and Table 2).

Additionally, the TG contents of fresh serum samples were evaluated employing three ranges: <50, 50–150, and >150 mg/dL. Table 1 shows the sample sizes for these ranges regarding the NMR and RLP groups. The distribution of the sample size showed a slight difference between groups. In the correlation analysis between groups for each level range, correlation coefficients for <50, 50–150, and >150 mg/dL TG were 0.48, 0.95, and 0.96, respectively (Table 2). According to these results, <50 mg/dL TG values in RLP were fairly correlated with NMR, while RLP measurements were strongly correlated with NMR in the 50–150 and >150 mg/dL TG ranges in the same individuals. In addition, the *p*-values of correlation for the <50, 50–150, and >150 mg/dL TG ranges were <0.0001. Scatter plots for correlations among all level ranges are demonstrated in Figure 1B–D.

The changes in TC values between RLP and NMR were assessed using 17,337 fresh serum samples. According to the Shapiro–Wilk test, TC values in NMR and RLP were not normally distributed, with a *p*-value of <0.001. The statistical significance of the variations between the NMR and RLP-derived levels was determined using *t*-tests with a *p*-value of <0.0001. Furthermore, Spearman analysis indicated a very strong correlation between RLP and NMR group contents (r: 0.96, *p*-value: <0.0001) (Figure 1E and Table 2).

Furthermore, three level ranges (<92, 92–200, and >200 mg/dL) were used to assess the TC levels in fresh serum samples. Table 1 illustrates the sample size distribution of the RLP and NMR groups for these ranges. When evaluating this distribution, the sample size with >200 mg/dL TC for the same sample group was higher in NMR. A correlation analysis was performed for each level range of TC. Accordingly, TC values less than 92 from RLP had a weak correlation (r: 0.32) with NMR values (Figure 1F). In addition, the *p*-value was 0.2102768. The low r value may be associated with a small sample size for that range. The correlations for 92–200 and >200 mg/dL TC values between the RLP and NMR groups increased sharply and were moderate with r = 0.88 (Figure 1G,H, and Table 2).

**Figure 2:**
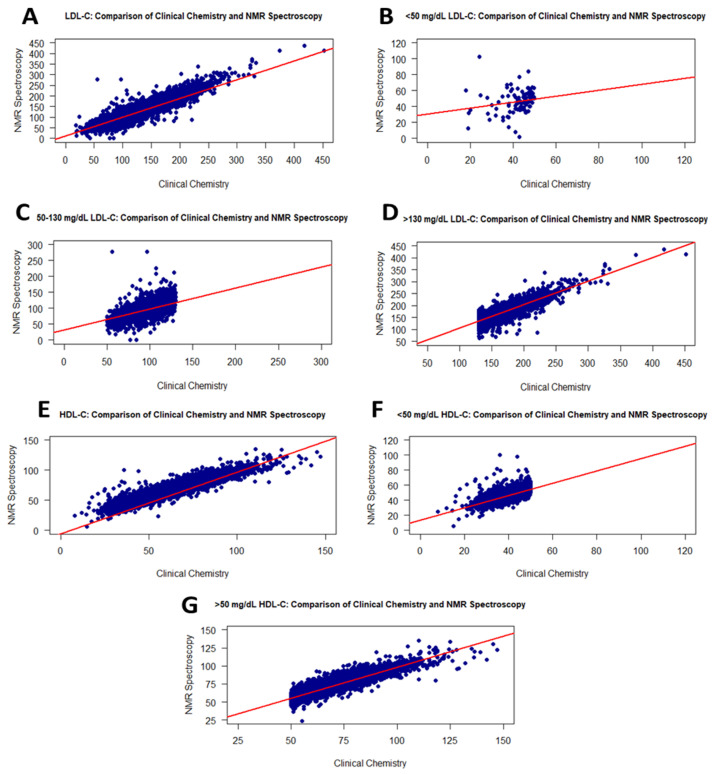
Graphs showing the correlation between the RLP and the NMR methods for LDL-C and HDL-C values. Scatter plots of correlations for all LDL-C and HDL-C values (**A**,**E**): <50 mg/dL LDL-C (**B**), 50–130 mg/dL LDL-C (**C**), >130 mg/dL LDL-C (**D**), <50 mg/dL HDL-C (**F**), and >50 mg/dL HDL-C (**G**).

LDL-C measurements of 17,337 fresh serum samples were evaluated to determine the association between the RLP and NMR methods. The LDL-C values measured with RLP were linearly associated with those of NMR, with r = 0.93 (Figure 2A). These values did not exhibit a normal distribution in any group. In addition, there was a significant difference between RLP and NMR according to the *t*-test (*p* < 0.0001) (Table 2). Moreover, Table 1 shows the sample size in each range for RLP and NMR. The LDL-C values of samples were distributed similarly between the two methods.

As shown in Figure 2, the <50 mg/dL LDL-C values in RLP had a weak correlation (r = 0.31) with NMR (Figure 2B), while the measured LDL-C values displayed a linear correlation with NMR within the range of 50–130 mg/dL, with an r value of 0.82 (Figure 2C). The higher LDL-C values exhibited a similar correlation (Figure 2D).

The HDL-C values of 17,337 individuals were measured using the NMR and RLP methods. The *p*-values for the Shapiro–Wilk test and *t*-test were <0.001 and <0.0001, respectively (Table 2). Figure 2 presents scatter plots illustrating the intergroup correlations which were very strong, with r = 0.95 (Figure 2E). When analyzing HDL-C levels at <50 or >50 mg/dL, the RLP- and NMR-derived values correlated with r = 0.77 (Figure 2F) and 0.90 (Figure 2G), respectively. Additionally, these correlations were statistically significant, with *p*-values of <0.0001. The group- and range-based distributions of HDL-C values are presented in Table 1.

### 3.2. Comparison Between RLP and NMR Analysis for TG, TC, HDL-C, and LDL-C in Frozen–Thawed Serum Samples

In the present study, fresh and frozen forms of 9559 serum samples were analyzed with the RLP and NMR methods. The fresh samples of the same individuals for TG, TC, HDL-C, and LDL-C levels were routinely analyzed with RLP, while their frozen samples were subjected to NMR lipid analysis. In the literature, it is reported that the repeated freeze–thaw process slightly affected lipid levels in serum samples. Furthermore, this study indicated strong relationships while employing RLP and NMR in fresh samples. In light of this observation, we present an NMR-based method to determine the impacts of freezing and thawing, which is quick and does not require the use of chemicals that could alter the sample composition. Therefore, the complementary use of NMR spectroscopy to support the routine biochemical analyses of frozen samples is demonstrated.

Table 3 shows the lipid distribution for each group. Additionally, the statistical analysis results are provided in Table 2.

**Figure 3:**
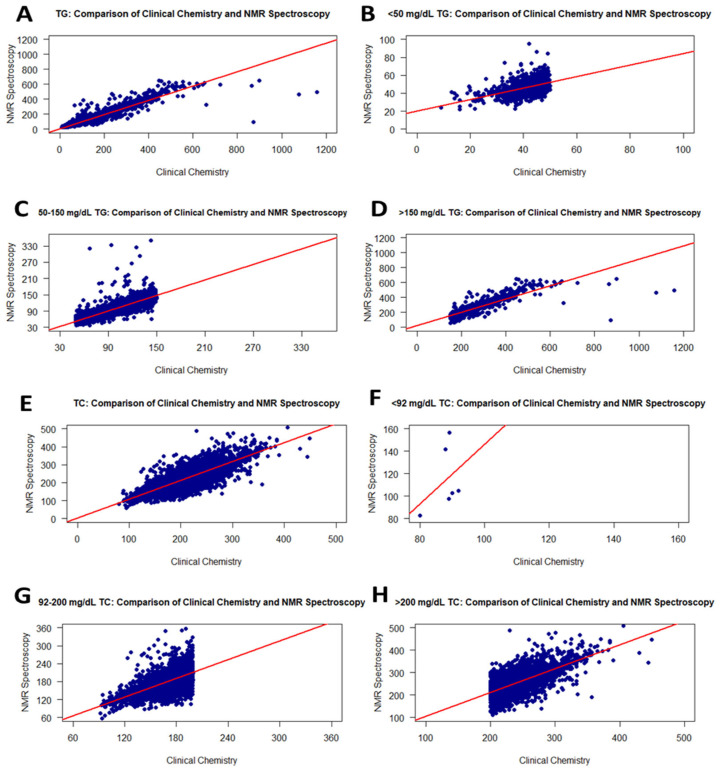
Graphs showing the correlation between RLP and the NMR method for TG and TC values on frozen samples. Scatter plots of correlations for all TG and TC values (**A**,**E**): <50 mg/dL TG (**B**), 50–150 mg/dL TG (**C**), >150 mg/dL TG (**D**), <92 mg/dL TC (**F**), 92–200 mg/dL TC (**G**), and >200 mg/dL TC (**H**).

TG measurements were performed on fresh serum samples using RLP and on frozen serum samples using NMR. When comparing TG levels measured by RLP and NMR for the same samples, no significant difference was found, with a *t*-test *p*-value of 0.4361696. Additionally, the data for each group were not normally distributed. The RLP-derived TG levels exhibited a strong positive association with those from NMR (r = 0.97), indicating that the freeze–thaw process consistently did not have any adverse effects across a wide range of TG contents (<50, 50–150, >150 mg/dL). Specifically, the TG values within these ranges showed correlation coefficients of 0.50, 0.93, and 0.89, respectively, similar to the correlation with the TG levels of fresh samples described in Section 3.1 (Table 2). The scatter plots illustrating these correlations are presented in Figure 3A–D. Additionally, the distribution of TG values within each group is provided in Table 3.

For the TC levels in 9559 serum samples, the correlation between RLP (fresh samples) and NMR (frozen) was moderate (r = 0.84). However, the correlation slightly decreased from 0.96 to 0.84 compared to the analysis (NMR and RLP) performed with fresh samples. Figure 3E–H show the correlation curve of TC values from RLP and NMR. Additionally, in the *t*-test performed on the obtained data, it was determined that both analysis methods show a significant difference. The *p*-value for the normal distribution was <0.0001. When the groups were separated into three TC level ranges, levels <92 mg/dL TC were weakly correlated with r = 0.37 (Figure 3F and Table 2), while TC levels of 92–200 and >200 mg/dL were fairly correlated with r = 0.68 (Figure 3G and Table 2) and r = 0.69 (Figure 3H and Table 2), respectively. Thus, the freezing process affects increasing TC levels quite negatively compared to fresh samples.

**Figure 4:**
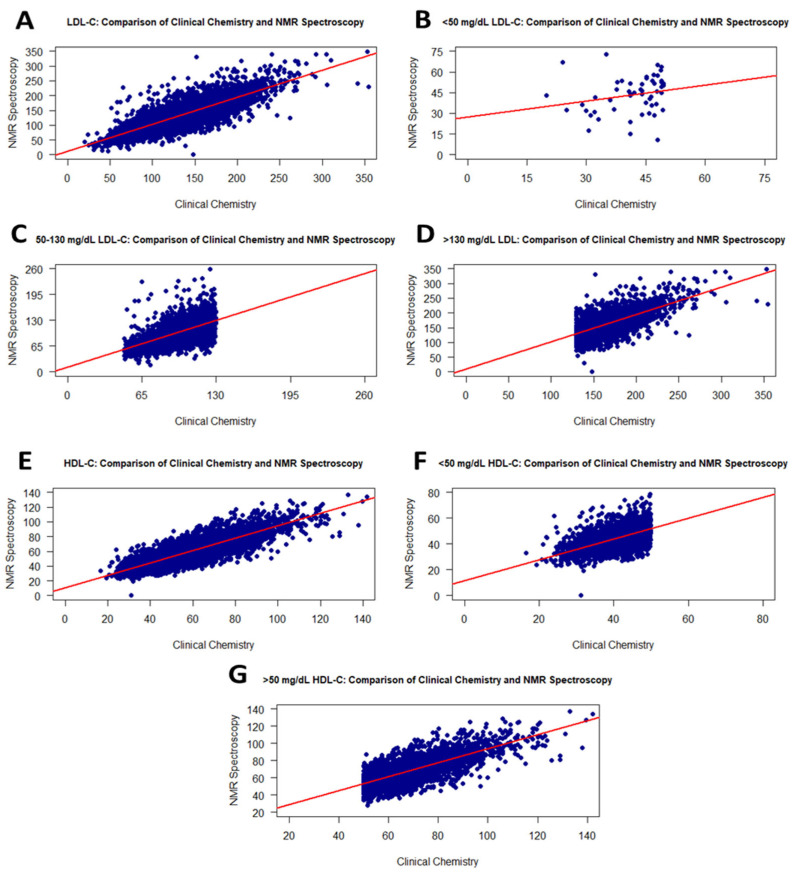
Graphs showing the correlation between RLP and the NMR method for LDL-C and HDL-C values on the frozen samples. Scatter plots of correlations for all LDL-C and HDL-C values (**A**,**E**): <50 mg/dL LDL-C (**B**), 50–130 mg/dL LDL-C (**C**), >130 mg/dL LDL-C (**D**), <50 mg/dL HDL-C (**F**), and >50 mg/dL HDL-C (**G**).

Due to the effect of the freezing process, the correlation of LDL-C levels slightly decreased from 0.93 to 0.83 compared to the fresh samples. Moreover, the correlation analysis for LDL-C values is statistically significant. When investigating level ranges, LDL-C levels <50 mg/dL in fresh samples showed a weak correlation with an r value of 0.33 compared with the frozen samples. Correlation coefficients for LDL-C levels of 50–130 mg/dL and >130 mg/dL increased to 0.66 and 0.65, respectively (Table 2). The correlation curves are shown in Figure 4A–D. According to the *t*-test analysis, there was no significant difference with a *p*-value of 0.4154 between the 9559 fresh and frozen serum samples (Table 2).

HDL-C levels from RLP showed a moderate correlation (r = 0.86) with HDL-C values from NMR using frozen samples, and the analysis was statistically significant (Figure 4E). Furthermore, a significant difference was observed when comparing HDL-C levels of serum samples by group using a *t*-test. While samples showed a positive correlation of 0.61 for HDL values less than 50 mg/dL, a correlation of 0.73 was determined for HDL values greater than 50 mg/dL (Figure 4F,G).

## 4. Discussion

This study is the first to compare the RLP and NMR methods for quantitatively analyzing TG, TC, HDL-C, and LDL-C using a large sample size of the Turkish population.

As is well known, the methods used for lipid parameters in clinical chemistry are considered standardized and validated. In this study, the analytical performance characteristics of the RLP and NMR methods were compared to demonstrate the accuracy, reliability, and usability of the NMR method. NMR measurement results were examined through validation against the gold standard, correlation studies, and response studies under different sample conditions. When compared with clinical methods in the literature, comparative studies based on the advantages offered by the NMR method have demonstrated that it is an alternative and reliable method, particularly for the analysis of lipid parameters. An atlas for NMR-based metabolic biomarker profiling by Julkunen et al. comparing clinical chemistry measurements with NMR measurements of biomarkers, including lipid parameters, was presented for various diseases using 118,461 plasma samples from the UK Biobank [16]. The study demonstrated high concordance between disease associations for eight biomarkers measured using both NMR and clinical chemistry. Consistency between NMR-based and clinical chemistry analyses in absolute concentrations was demonstrated. A study of this magnitude demonstrates the accuracy and validity of NMR data [16]. Furthermore, a joint consensus panel from the European Atherosclerosis Society (EAS) and the European Federation of Clinical Chemistry and Laboratory Medicine (EFLM) stated that the use of NMR and LC-MS/MS should be standardized and validated due to the inadequacy of routine clinical chemistry practices for Apo-B quantification, LDL-P, and VLDL count and size [17].

In the current study, RLP and NMR methods produced similar results based on the correlation analysis between the main lipids (TC, TG, HDL-C and LDL-C). This demonstrates the comparability of the methods used to assess lipids. Correlation analyses of RLP and NMR-derived values, particularly with fresh samples, yielded a high correlation between 0.93 and 0.98. Studies comparing standard lipid analyses with the NMR method are quite limited. On the other hand, Rief et al. compared two different NMR methods (lipoprofile and lipofit) with clinical chemistry-based lipid analysis [18]. In the results of the study, correlation coefficients between the standard lipid method and lipoprofile NMR method for TC, TG, LDL-C, and HDL-C values were 0.964, 0.979, 0.941, and 0.837, respectively. These results were similar to the comparative findings of the standard lipid and the lipofit NMR methods. Another correlation study was performed using standard clinical analysis and NMR measurements. The total TG, total TC, HDL-C, and LDL-C correlation coefficients were 0.99, 0.98, 0.88, and 0.97, respectively [19]. NMR spectroscopy has recently been investigated as an alternative to biochemical lipid tests commonly utilized in clinical settings. NMR not only indicates the cholesterol content in lipoprotein structures, as with RLP, but also indicates the number of lipoprotein particles. The literature indicates a stronger correlation between LDL-P levels and CVD, particularly when discrepancies are observed in LDL-C. Therefore, a number of expert panels now advise taking LDL-P levels into account when making decisions and directing medical treatment [8,20,21]. In this context, this study shows that NMR spectroscopy is a unique approach compared to RLP in clinical applications.

Furthermore, in the current study, when lipids were separated according to normal or risky level ranges, the values with lower levels for all lipid structures had lower correlation coefficients. This decrease in correlation may be due to the low sample size. Although the correlation is not directly affected by the sample size, the reliability, validity, and significance are statistically affected. Since deviations in data decrease as the sample size increases, correlation coefficients converge to true values [22]. In addition, although there was a strong association between RLP and NMR levels, a significant difference was determined through a *t*-test. In RLP and NMR measurements, *p*-values in the *t*-test may be significant due to the very low standard deviations within the group. This high significance level shows that both methods have very good performance in lipid analysis. According to this, while a very high correlation was observed between both methods for a particular lipid parameter, the significance level for the *t*-test was determined to be high due to the low standard deviation values that the methods have within themselves. This trend is clearly seen in LDL-C analysis. As the LDL-C concentration increased from <50 mg/dL to >150 mg/dL in both fresh and frozen samples, the correlation increased from 0.31 to 0.82, and the significance level in the *t*-test increased from 0.004029 to 1.970015 × 10^−177^ (very small values are defined as <0.0001 in Table 2). This shows that increasing the LDL-C concentration and sample size will increase the measurement performance of the method (due to the detection limit of the method/device), and the significance level in the *t*-test will increase. Correlation values also increased. Conversely, the opposite trend is observed for total TG values. As the TG concentration increases, the decrease in the *t*-test significance level despite the increased correlation value indicates that the standard deviation of either the NMR or RLP methods is high, and this value affects the *t*-test. Furthermore, as the sample size increases, even small differences between the averages of the measurements (such as 1–2 mg/dL) lower the *p*-value and cause them to appear statistically significant due to the nature of statistical tests [23,24].

The literature shows that small changes can occur in lipid profile concentrations in serum samples due to preanalytical processes such as freezing and thawing. Many studies have focused on freeze–thaw cycles and temperatures. Wang et al. examined changes in lipid and metabolite concentrations in serum and urine samples obtained from a total of 40 donors across multiple freeze–thaw cycles using NMR spectroscopy [9]. The study found no significant difference in urine metabolite concentrations, while minor changes were observed in serum lipoprotein and metabolite levels. Additionally, Cuhadar et al. reported no significant change in lipoprotein values after ten freeze–thaw cycles in serum samples obtained from 15 patients [25]. Abraham et al. observed that HDL and cholesterol levels in 70 subjects varied significantly after a single freeze–thaw cycle, whereas LDL and TG levels remained stable [26].

Based on the studies mentioned above, the other part of the present study examined the changes in TG, TC, HDL-C, and LDL-C levels after a single freeze–thaw cycle. In the first part of the current study, correlation studies between standard RLP and NMR analyses conducted in fresh samples indicated a strong correlation. Based on this result, an approach using NMR analysis, performed rapidly and without the use of reagents that could affect sample contents, has been proposed to determine the effect of freezing and thawing. In studies performed with frozen samples, the correlation between lipid structures, except for TG, decreased to r < 0.87. This decline may be due to the thawing conditions of the samples. It is stated in the literature that compared to thawing in the cold, thawing at room temperature had a smaller impact [27]. However, compared to the literature, the lipid values observed in this work showed a more significant difference. Accordingly, it has been shown that in laboratories where standard RLPs are performed, NMR lipid analyses of samples in biobanks after freezing may have a lower correlation and a lower reliability level. In this context, in the current study, good correlation was found for certain lipid targets at certain concentrations, but it should not be ignored that there is no good correlation or accuracy in frozen samples. To clarify the impact of freezing on both methods and help identify which approach is most reliable, further comparative studies are needed.

## 5. Conclusions

This study compared the TG, TC, HDL-C, and LDL-C levels of 17,337 fresh and 9559 frozen serum samples, analyzed using RLP and NMR. Strong correlations were observed between the TG (r: 0.98), TC (r: 0.96), HDL-C (r: 0.95), and LDL-C (r: 0.93) contents ascertained employing the two methods. Furthermore, the use of NMR spectroscopy to support routine biochemical analyses in frozen samples resulted in slightly lower correlation coefficients. Therefore, although the NMR approach for lipid analysis is a unique alternative method for fresh samples, it has not demonstrated high efficiency for frozen samples. The correlation coefficients of TC, LDL-C and HDL-C from frozen samples decreased from 0.96 to 0.84, from 0.93 to 0.83, and from 0.95 to 0.86, respectively. However, considering the advantages of NMR, it is anticipated that it could be preferred as an alternative method with the development of a specific error rate formulation.

## Figures and Tables

**Table 1:** Distribution of sample sizes according to the range of lipid levels in fresh serum samples for each group.

	Cholesterol (mg/dL)			
	<92	92–200		>200
RLP (*n*) *	17	8711		8609
**NMR (*n*)**	8	5921		11,408
	**Triglyceride (mg/dL)**			
	**<50**	**50–150**		**>150**
**RLP (** ***n*** **)**	1650	11,765		3919
**NMR (** ***n*** **)**	1381	11,935		4018
	**HDL-C (mg/dL)**			
	**<50**		**>50**	
**RLP (** ***n*** **)**	7308		10,029	
**NMR (** ***n*** **)**	4996		12,341	
	**LDL-C (mg/dL)**			
	**<50**	**50–130**		**>130**
**RLP (** ***n*** **)**	96	9041		8200
**NMR (** ***n*** **)**	127	8055		9155

* *n*: sample size.

**Table 2:** Summary of statistical analysis in comparison with the RLP and NMR methods.

		RLP vs. NMR on Fresh Serum Samples			RLP vs. NMR on Frozen Serum Samples *		
	Level Range	Correlation Coefficient (r)	Correlation *p*-Value	*t*-Test *p*-Value	Correlation Coefficient (r)	Correlation *p*-Value	*t*-Test *p*-Value
**TG (mg/dL)**	**Total**	0.98	<0.0001	<0.0001	0.97	<0.0001	0.4361696
	**<50**	0.48	<0.0001	<0.0001	0.50	<0.0001	<0.0001
	**50–150**	0.95	<0.0001	<0.0001	0.93	<0.0001	0.005372
	**>150**	0.96	<0.0001	<0.0001	0.89	<0.0001	0.113
**TC** **(mg/dL)**	**Total**	0.96	<0.0001	<0.0001	0.84	<0.0001	<0.0001
	**<92**	0.32	0.2102768	0.0001154	0.37	0.497	0.063
	**92–200**	0.88	<0.0001	<0.0001	0.68	<0.0001	<0.0001
	**>200**	0.88	<0.0001	<0.0001	0.69	<0.0001	<0.0001
**LDL-C** **(mg/dL)**	**Total**	0.93	<0.0001	<0.0001	0.83	<0.0001	0.4154
	**<50**	0.31	0.00217	0.004029	0.33	0.0151	0.334
	**50–130**	0.82	<0.0001	<0.0001	0.66	<0.0001	<0.0001
	**>130**	0.82	<0.0001	<0.0001	0.65	<0.0001	<0.0001
**HDL-C** **(mg/dL)**	**Total**	0.95	<0.0001	<0.0001	0.86	<0.0001	<0.0001
	**<50**	0.77	<0.0001	<0.0001	0.61	<0.0001	<0.0001
	**>50**	0.90	<0.0001	<0.0001	0.73	<0.0001	<0.0001

* Frozen forms of the same serum samples were measured with the NMR method.

**Table 3:** Distribution of sample sizes per range levels of lipids in frozen serum samples for each group.

	**Cholesterol (mg/dL)**			
	**<92**	**92–200**		**>200**
RLP * (*n* ^a^)	6	4505		5048
NMR ** (*n*)	13	3686		5860
	**Triglyceride (mg/dL)**			
	**<50**	**50–150**		**>150**
RLP * (*n*)	783	6581		2195
NMR ** (*n*)	683	6774		2102
	**HDL-C (mg/dL)**			
	**<50**		**>50**	
RLP * (*n*)	4003		5556	
NMR ** (*n*)	3494		6065	
	**LDL-C (mg/dL)**			
	**<50**	**50–130**		**>130**
RLP * (*n*)	54	4753		4752
NMR ** (*n*)	113	4787		4659

* The RLP method was performed on fresh serum samples. ** The NMR method was performed on frozen serum samples. ^a^ *n*: sample size.

## Data Availability

Due to ethical and privacy restrictions, the data generated or analyzed during this study are not publicly available but can be provided by the corresponding author upon reasonable request.

## Abbreviations

The following abbreviations are used in this manuscript:

RLP	Routine lipid profile
NMR	Nuclear magnetic resonance
CVD	Cardiovascular disease
TC	Total cholesterol
TG	Triglyceride
LDL	Low-density lipoproteins
LDL-C	LDL cholesterol
HDL	High-density lipoproteins
HDL-C	HDL cholesterol
LDL-P	Low-density lipoprotein particle number

## References

[1] Li M., Yang L., Bai Y., Liu H. (2014). Analytical Methods in Lipidomics and Their Applications. Anal. Chem..

[2] Teo C.C., Chong W.P.K., Tan E., Basri N.B., Low Z.J., Ho Y.S. (2015). Advances in Sample Preparation and Analytical Techniques for Lipidomics Study of Clinical Samples. TrAC Trends Anal. Chem..

[3] Bali S., Utaal M.S. (2019). Serum Lipids and Lipoproteins: A Brief Review of the Composition, Transport and Physiological Functions. Int. J. Sci. Rep..

[4] Bhargava S., de la Puente-Secades S., Schurgers L., Jankowski J. (2022). Lipids and Lipoproteins in Cardiovascular Diseases: A Classification. Trends Endocrinol. Metab..

[5] Kenneth R., Feingold M. (2024). Introduction to Lipids and Lipoproteins. https://www.ncbi.nlm.nih.gov/books/NBK305896/.

[6] Ahotupa M. (2024). Lipid Oxidation Products and the Risk of Cardiovascular Diseases: Role of Lipoprotein Transport. Antioxidants.

[7] Garcia E., Bennett D.W., Connelly M.A., Jeyarajah E.J., Warf F.C., Shalaurova I., Matyus S.P., Wolak-dinsmore J., Oskardmay D.N., Young R.M. (2020). The Extended Lipid Panel Assay: A Clinically- Deployed High-Throughput Nuclear Magnetic Resonance Method for the Simultaneous Measurement of Lipids and Apolipoprotein B. Lipids Health Dis..

[8] Matyus S.P., Braun P.J., Wolak-dinsmore J., Jeyarajah E.J., Shalaurova I., Xu Y., Warner S.M., Clement T.S., Connelly M.A., Fischer T.J. (2014). NMR Measurement of LDL Particle Number Using the Vantera ^®^ Clinical Analyzer. Clin. Biochem..

[9] Wang F., Debik J., Andreassen T., Euceda L.R., Haukaas T.H., Cannet C., Schäfer H., Bathen T.F., Giskeødegård G.F. (2019). Effect of Repeated Freeze-Thaw Cycles on NMR-Measured Lipoproteins and Metabolites in Biofluids. J. Proteome Res..

[10] Lee J., Young S., Shin S. (2015). Effect of Repeated Freezing and Thawing on Biomarker Stability in Plasma and Serum Samples. Osong Public Health Res. Perspect..

[11] Loo R.L., Lodge S., Kimhofer T., Bong S.H., Begum S., Whiley L., Gray N., Lindon J.C., Nitschke P., Lawler N.G. (2020). Quantitative In-Vitro Diagnostic NMR Spectroscopy for Lipoprotein and Metabolite Measurements in Plasma and Serum: Recommendations for Analytical Artifact Minimization with Special Reference to COVID-19/SARS-CoV-2 Samples. J. Proteome Res..

[12] Pinto J., Domingues M.R.M., Galhano E., Pita C., Do Céu Almeida M., Carreira I.M., Gil A.M. (2014). Human Plasma Stability during Handling and Storage: Impact on NMR Metabolomics. Analyst.

[13] Wist J., Nitschke P., Conde R., de Diego A., Bizkarguenaga M., Lodge S., Hall D., Chai Z., Wang W., Kowlessur S. (2025). Benchtop Proton NMR Spectroscopy for High-Throughput Lipoprotein Quantification in Human Serum and Plasma. Anal. Chem..

[14] Akoglu H. (2018). User’s Guide to Correlation Coefficients. Turkish J. Emerg. Med..

[15] Kwak S.G., Park S.H. (2019). Normality Test in Clinical Research. J. Rheum. Dis..

[16] Julkunen H., Cichońska A., Tiainen M., Koskela H., Nybo K., Mäkelä V., Nokso-Koivisto J., Kristiansson K., Perola M., Salomaa V. (2023). Atlas of Plasma NMR Biomarkers for Health and Disease in 118,461 Individuals from the UK Biobank. Nat. Commun..

[17] Nordestgaard B.G., Langlois M.R., Langsted A., Chapman M.J., Aakre K.M., Baum H., Borén J., Bruckert E., Catapano A., Cobbaert C. (2020). Quantifying Atherogenic Lipoproteins for Lipid-Lowering Strategies: Consensus-Based Recommendations from EAS and EFLM. Atherosclerosis.

[18] Rief M., Raggam R., Rief P., Metnitz P., Stojakovic T., Reinthaler M., Brodmann M., März W., Scharnagl H., Silbernagel G. (2022). Comparison of Two Nuclear Magnetic Resonance Spectroscopy Methods for the Measurement of Lipoprotein Particle Concentrations. Biomedicines.

[19] Bathen T.F., Krane J., Engan T., Bjerve K.S., Axelson D. (2000). Quantification of Plasma Lipids and Apolipoproteins by Use of Proton NMR Spectroscopy, Multivariate and Neural Network Analysis. NMR Biomed..

[20] Contois J.H., McConnell J.P., Sethi A.A., Csako G., Devaraj S., Hoefner D.M., Warnick G.R. (2009). Apolipoprotein B and Cardiovascular Disease Risk: Position Statement from the AACC Lipoproteins and Vascular Diseases Division Working Group on Best Practices. Clin. Chem..

[21] Davidson M.H., Ballantyne C.M., Jacobson T.A., Bittner V.A., Braun L.T., Brown A.S., Brown W.V., Cromwell W.C., Goldberg R.B., McKenney J.M. (2011). Clinical Utility of Inflammatory Markers and Advanced Lipoprotein Testing: Advice from an Expert Panel of Lipid Specialists. J. Clin. Lipidol..

[22] Schönbrodt F.D., Perugini M. (2013). At What Sample Size Do Correlations Stabilize?. J. Res. Pers..

[23] Cohen J. (1988). Statistical Power Analysis for the Behavioral Sciences.

[24] Kim T.K., Park J.H. (2019). More about the Basic Assumptions of T-Test: Normality and Sample Size. Korean J. Anesthesiol..

[25] Cuhadar S., Koseoglu M., Atay A., Dirican A. (2013). The Effect of Storage Time and Freeze-Thaw Cycles on the Stability of Serum Samples. Biochem. Medica.

[26] Abraham R.A., Rana G., Agrawal P.K., Johnston R., Sarna A., Ramesh S., Acharya R., Khan N., Porwal A., Kurundkar S.B. (2021). The Effects of a Single Freeze-Thaw Cycle on Concentrations of Nutritional, Noncommunicable Disease, and Inflammatory Biomarkers in Serum Samples. J. Lab. Physicians.

[27] Rebholz S.L., Melchior J.T., Welge J.A., Remaley A.T., Davidson W.S., Woollett L.A. (2017). Effects of Multiple Freeze/Thaw Cycles on Measurements of Potential Novel Biomarkers Associated With Adverse Pregnancy Outcomes. J. Clin. Lab. Med..

